# Structured knowledge representation of the South China Sea: An LLM-based knowledge graph approach

**DOI:** 10.1371/journal.pone.0351132

**Published:** 2026-06-11

**Authors:** Ruinan Zhao, Zhifan Han, Huiling Liu

**Affiliations:** 1 School of Foreign Languages, Guangzhou Maritime University, Guangzhou, Guangdong, China; 2 School of Architectural Engineering, Guangzhou Institute of Science and Technology, Guangzhou, Guangdong, China; Guilin University of Technology, CHINA

## Abstract

The South China Sea (SCS) presents a significant research challenge due to severe data fragmentation and semantic heterogeneity across disparate historical, legal, and geopolitical sources. Conventional linear research approaches often fail to systematically integrate these disparate records. This study develops and demonstrates an automated LLM-driven framework for constructing the South China Sea Knowledge Graph (SCS-KG). The three-stage process (extraction, normalization, and verification) converts heterogeneous textual sources into a coherent, machine-readable structure that integrates the region’s historical, cultural, and geopolitical dimensions. After verification, the SCS-KG comprises 59,836 entities and 652,018 triples. The resulting knowledge graph supports three analytical applications: (1) evidence-based query answering that synthesizes facts across centuries (e.g., from ancient textual records to modern legal declarations); (2) discovery of implicit, multi-hop relationships linking state-level governance and micro-level social practices; and (3) rapid, entity-centric profiling of complex geopolitical instruments. This LLM-based approach provides a replicable model for structured knowledge representation and enables integrated, evidence-based analysis in South China Sea studies.

## 1. Introduction

The South China Sea (SCS) is a region of critical geopolitical and historical importance, characterized by complex territorial disputes and a vast, multi-disciplinary knowledge domain spanning law, history, and international relations [1,2]. Despite increasing digitalization efforts, existing resources such as the *Nanhai Library Digital Resource Repository* developed by Nanjing University (http://202.119.47.101/nh_view/) and the *Digital Nanhai* curated by the National Institute for South China Sea Studies (https://csarc.nanhai.org.cn/home) remain fragmented and lack semantic interoperability [3,4].

Knowledge graphs (KGs), typically represented as subject–predicate–object triples, serve as structured and explicit representations of factual knowledge [5–7]. They are essential in knowledge-intensive tasks and information retrieval, where unstructured data can be transformed into machine-interpretable formats. KGs have been applied in various domains, including medicine, biology, social networks, and higher education [8–10]. However, traditional KG construction methods, such as natural language processing (NLP) pipelines and rule-based systems, often rely on manual annotation and domain expertise, making them time consuming and limiting their scalability in practice [11]. These challenges are particularly pronounced in the South China Sea context, where cross-disciplinary data spanning geography, history, maritime law, and international relations must be integrated coherently.

Recent advances in large language models (LLMs) have opened new avenues for KG construction by addressing key limitations inherent in traditional methods. Unlike rule-based systems or early NLP approaches that rely heavily on manual annotation and predefined rules, LLMs which are trained on massive textual corpora possess extensive world knowledge and can emulate the reasoning capacity of domain experts [12]. LLMs also distinguish themselves by resolving semantic ambiguities and inferring implicit relationships, both of which commonly hinder the precision of conventional KG pipelines [5,13]. Moreover, their few-shot and zero-shot learning capabilities substantially reduce reliance on large annotated datasets, an especially valuable feature for under-resourced domains such as maritime historical research, where labeled data remain limited [14].

Building on these advantages, this study develops a domain-specific implementation of an LLM-assisted knowledge graph framework for constructing the South China Sea Knowledge Graph (SCS-KG). The three-stage pipeline integrates (1) **entity and relation extraction** using LLM-based identification of key information from textual sources, (2) **entity and relation normalization** through embedding and clustering to merge synonymous terms and reduce redundancy, and (3) **triple verification** using LLMs to assess factual correctness and assign confidence scores. This systematic design directly addresses this fundamental domain challenge, transforming fragmented, multi-source data into a coherent and verifiable knowledge representation. This study’s primary contribution is the validation of SCS-KG as a domain-specific analytical resource for South China Sea studies, moving beyond simple document retrieval to enable new forms of computational analysis.

Specifically, the contributions of this work are threefold:

- The development of a large-scale, machine-readable knowledge graph for the South China Sea that integrates fragmented historical, legal, and geopolitical information into a unified representation.
- The validation of this KG’s reliability for researchers through a multistage normalization and verification process, which specifically addresses the core domain challenges of semantic heterogeneity and data inconsistency.
- The demonstration of a computationally-assisted research workflow for SCS studies. We empirically show that the SCS-KG can answer complex, interdisciplinary queries by synthesizing facts across centuries, discover implicit multi-hop relationships (e.g., between state governance and social practices), and perform rapid entity-centric profiling-capabilities that are difficult to achieve efficiently with traditional, linear research methods.

## 2. Related work

### 2.1. The South China Sea in the digital age

The digital humanities field represents a fundamental interdisciplinary convergence, uniting traditional humanistic inquiry including history, literature, and cultural studies with rigorous computational methodologies [15,16]. This computational shift enables scholars to leverage data analysis techniques to interpret humanistic data, revealing latent patterns, themes, and connections within large, previously intractable datasets [17]. Central to this transformation is the Knowledge Graph (KG), a crucial data structure that integrates information from disparate sources, transforming current resources into representations interpretable by both machines and humans [8]. While general-purpose KGs such as Wikidata and DBpedia provide broad coverage, they often lack the necessary semantic depth and domain specificity required for complex applications, leading to fragmentation and insufficient representation in specialized research areas [9,18]. Consequently, a growing research trend focuses on leveraging machine learning (ML) and natural language processing (NLP) to construct domain-specific knowledge graphs across fields such as law, cultural heritage, and political science, enabling the accurate modeling of highly specialized knowledge [17,19]. Recent advancements in AI-assisted knowledge representation, semantic modeling, and structured information extraction have further strengthened this trend, particularly through hybrid machine learning and deep learning frameworks combined with explainable AI techniques. These developments demonstrate the potential of automated yet interpretable methods for handling complex, heterogeneous data in high-stakes domains [20–22].

The South China Sea (SCS) region is a critical academic and geopolitical subject, characterized by complex territorial disputes, rich natural resources, and immense strategic significance [23]. Research on the SCS is inherently multifaceted, spanning international relations, law, security, history, and culture [24]. The necessity of understanding the interconnections between these diverse disciplinary facets has driven a growing emphasis on interdisciplinary approaches [25]. Although historical and social science perspectives traditionally dominate this research, there is an increasing adoption of computational methods to achieve the digitization of research in this area [26].

Recent computational efforts focused on the SCS have primarily aimed to structure and analyze complex data. For instance, Wang et al. [4] focused on constructing a knowledge map of documentary evidence for rights protection by transforming raw data into a structured layer of evidentiary entries. Other studies have applied advanced ML to classification tasks, such as Peng et al. [27], who proposed a BERT-based system for fine-grained, multi-label classification of evidentiary data to reveal latent relationships. Furthermore, specialized NLP has been crucial in dealing with resource-scarce historical texts, as seen in the work of Wei et al. [26], who developed an ALBERT-based model to enhance toponymic entity recognition in the Genglubu text. Similarly, Peng and Li [3] have employed social network analysis (SNA) to quantify the temporal dynamics of geopolitical interactions, offering a data-driven framework for regional relations.

While these pioneering studies have significantly advanced the innovation and modernization of SCS knowledge representation within the context of artificial intelligence, several critical gaps remain. Compared to digital resources available in the natural sciences, research on the creation of integrated digital infrastructure in the humanities and social sciences within this region remains notably limited [28]. Furthermore, most existing computational studies employ either specialized, semi-automated methods that require substantial manual effort or focus on solving isolated NLP tasks, which collectively reduces overall efficiency and scope. Crucially, the fundamental challenge of multiplicity and heterogeneity across diverse data sources including historical records, legal documents, and cultural artifacts continues to impede comprehensive analysis via current sequential NLP methodologies. Addressing such heterogeneity requires pipeline architectures that incorporate principled normalization, feature verification, and interpretability mechanisms—design considerations that remain active challenges across AI-driven structured information extraction tasks [22,29,30]. This paper addresses these limitations by developing the SCS-KG, designed to provide a cohesive, structured, and interoperable resource that enables multi-disciplinary discovery and analytical reasoning across fragmented historical, cultural, and geopolitical narratives.

### 2.2. LLM-based knowledge graph construction

Knowledge Graphs (KGs) are fundamental for organizing and representing information in a structured, machine-interpretable format. Traditional methods for KG construction including manual curation, rule-based systems, and classical machine learning (ML)/natural language processing (NLP) approaches, have proven effective but face inherent limitations [12,13]. Specifically, these methods often struggle with contextual reasoning and exhibit poor adaptability to novel or complex information due to their reliance on rigid, predefined rules and categories [11]. This structural rigidity hinders their performance when scaling to diverse, high-volume, and heterogeneous datasets that characterize modern domains [14].

Recent advancements in Large Language Models (LLMs) offer a promising solution to these challenges, supporting more flexible approaches to knowledge extraction, representation, and reasoning. LLMs facilitate various aspects of KG construction by leveraging their superior ability to process and generate natural language, enabling robust zero-shot extraction of entities, relationships, and attributes [14]. Beyond simple information extraction, LLMs show strong reasoning performance through in-context learning (ICL), reducing the need for labeled datasets. Methods for integrating LLMs range from zero-shot prompting [31], where the task is reframed as a multi-turn Q&A (e.g., ChatIE), to more structured approaches like KICGPT [32], which integrates LLMs with traditional triple-based retrievers for knowledge graph completion. While training-free methods such as ICL and prompt engineering are efficient, other studies emphasize the value of fine-tuning to enhance structural awareness, arguing that structural embeddings contain richer semantic information than text-based prompts [33]. The reliability of automated knowledge extraction pipelines is further contingent on the integration of interpretability mechanisms and multi-stage verification strategies, which improve the semantic validity of extracted content and reduce noise introduced during automated processing [21,29].

Despite these rapid advancements, the core limitations of LLM outputs remain a critical challenge, notably regarding accuracy and reliability due to hallucinations and contextual misunderstandings [34,35]. This challenge has motivated the development of robust hybrid frameworks that integrate LLMs with external knowledge and verification systems to bolster credibility. Approaches like KGGen [5] and SAC-KG [11] utilize multi-stage methodologies involving generator, verifier, and pruner components to mitigate graph noise, incompleteness, and knowledge hallucination. In these frameworks, the LLM is treated as a domain expert operating under a structured governance layer. The importance of such governance layers is further underscored by recent findings showing that pipeline designs incorporating structured normalization, feature pruning, and explainability stages consistently yield more robust and trustworthy outputs in automated knowledge extraction tasks [20,22].

Despite these documented advancements, a significant domain-specific gap remains: to the best of our knowledge, no prior work has constructed a comprehensive KG focused on the complex, heterogeneous South China Sea domain, nor applied advanced LLM-integrated techniques to knowledge extraction and validation in this critical geopolitical and historical context. The present framework addresses this gap while differing from existing approaches in three respects that are particularly salient for this domain. First, it targets open-ended entity-relation extraction from heterogeneous historical, legal, geopolitical, and cultural texts rather than classification or detection tasks. Second, it combines LLM-based extraction with embedding-based normalization and LLM-assisted triple verification to address semantic redundancy and factual noise within a single integrated pipeline. Third, it evaluates the resulting KG not only through extraction metrics but also through domain-specific reasoning scenarios, positioning it as a domain-adapted KG construction workflow rather than a general explainable AI classifier.

## 3. Method

**Fig 1:**
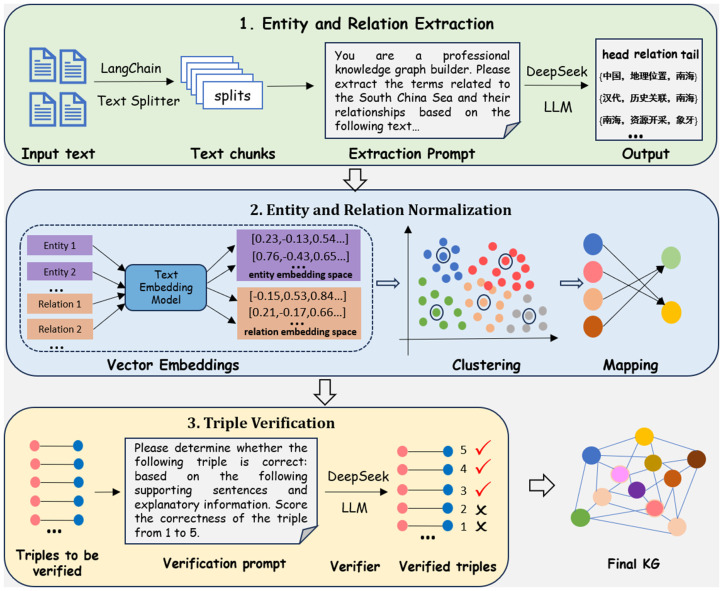
Pipeline overview of the framework for constructing the SCS-KG.

In this section, we provide an overview of the overall construction process for the SCS-KG, as illustrated in Fig 1. The pipeline is designed to transform raw textual data into a structured and verified knowledge graph. It consists of three key steps, which are **entity and relation extraction** (Section 3.1), **entity and relation normalization** (Section 3.2), and **triple verification** (Section 3.3).

### 3.1. Entity and relation extraction

The extraction of entities and relations uses LLMs to identify and extract entities along with their corresponding relations from natural language and directly outputs them. Given LLMs’ proficiency in language comprehension, the Generative Relation Extraction method is adopted [6]. However, lengthy documents often pose significant challenges due to LLMs’ limited context length. To address this, the original document is first split into smaller, more manageable chunks to fit within the LLM context window and at the same time preserve the semantic integrity of the text [34]. Each chunk is then analyzed by the LLM to generate a structured representation of the information contained within it, which includes the identification of key entities and their relations as well as the provision of their supporting sentences and explanations. The LLM is also prompted to label an entity type to the extracted entity from a predefined set of entity categories. The predefined entity categories are based on previous studies on named entity recognition (NER) with modifications to better fit current data [10,32]. By indicating what specific category the extracted entity belongs to, we can easily find out whether there are misclassifications of the entities by the LLM [32]. We impose strong constraints on the output format of the LLM via the Langchain OutputParser to ensure the output format (in JSON) of LLM. The prompt for the entity and relation extraction and the expected output format are illustrated in Table 1.

**Table 1:** Entity and relation extraction prompt and output format.

Entity and Relation Extraction Prompt	Expected Output Format
You are a professional knowledge graph builder. Please extract South China Sea-related entities (countries, geographical formations, resources, sea areas, events, agreements, organizations, etc.) and their relations (sovereignty, location, resource extraction, event association, agreement signing, etc.) from the text. **Output**: • List triples with supporting sentences • Explain relationship rationale • Label entities via categories: PER (person/ethnic groups), ORG (organizations), LOC (locations), TIM (time), QTY (quantity), EVT (events/laws), CUL (culture), BUS (trade), TEC (technologies), MR (marine resources) **Format**: • **Head**: {head} • **Relation**: {relation} • **Tail**: {tail} • **Supporting Sentence**: {supporting_sentence} • **Explanation**: {explanation} Ensure text-based content, accurate labels. And reasonable explanations.	{ “**head**”: “南海(South China Sea)”, “**tail**”: “中国技术(Chinese technology)”, “**head type**”: “LOC”, “**tail type**”: “TEC”, “**relation**”: “技术推动(technology promotion)”, “**supporting sentence**”: “在历史上，以中国技术为主要推动力的探索行为，经历了一个在南海上“画弧成圈” 的过程。(Historically, exploration activities driven primarily by Chinese technology have undergone a process of drawing arcs into circles in the South China Sea.)”, “**explanation**”: “中国技术推动了南海上的探索行为，与技术推动相关。(Chinese technology has promoted exploration activities in the South China Sea, which is related to technology promotion.)”, }

### 3.2. Entity and relation normalization

After extracting the entities and relations from the source texts, we may have duplicate or synonymous entities and redundant relations. The normalization process aims to merge the extracted entities and relations which have the same underlying meaning, e.g., “海南 (Hainan)” might be clustered with “海南岛 (Hainan Island)” and “贸易往来 (trade exchanges)” with “贸易联系 (trade connections)”, “贸易交流 (trade communications)”, “商贸往来 (commercial exchanges)”. This reduces the likelihood of semantically similar duplicate entities or relationships and prevents the formation of sparse KGs. The normalization process involves several steps, including embedding, clustering, and mapping. It helps to create a more accurate and eﬀicient knowledge graph by reducing redundancy and standardizing entities and relations.

In our approach, the nodes and edges are first vectorized into numerical vectors using the text-embedding-3-large model. Once the embeddings are obtained, similar entities and relations are grouped via hierarchical clustering using the average linkage method, with cosine similarity as the similarity measure (see Equation 1 below):


$$\begin{array}{c} \begin{array}{cc} & cosine\;similarity=\frac{\overset{\to }{A}\cdot \overset{\to }{B}}{∥\overset{\to }{A}∥\cdot ∥\overset{\to }{B}∥}=\frac{\sum_{i=\text{1}}^{n}A_{i}B_{i}}{\sqrt{\sum_{i=\text{1}}^{n}\overset{\text{2}}{\underset{i}{A}}}\cdot \sqrt{\sum_{i=\text{1}}^{n}\overset{\text{2}}{\underset{i}{B}}}} \end{array} \end{array}$$ (1)

where $\vec{A}$ and $\vec{B}$ denote two vectors, $A_{i}\;$and $B_{i}$ the *i*-th components, and *n* is the vector dimensionality. A cosine similarity value closer to 1 indicates a higher degree of similarity.

After clustering, a representative element for each cluster is determined. This representative is identified as the item exhibiting the highest similarity to the cluster’s weighted average embedding (weighted by the norm of each embedding vector), and serves as the canonical form for standardizing all cluster members.

### 3.3. Triple verification

Although normalization process effectively reduces redundancy and duplicate triples generated by the LLM, errors in the outputted triples may still persist. To further improve the quality of LLM-generated outputs, we introduce a Triple Verification step designed to identify and eliminate erroneous triples. All extracted triples are evaluated using the LLM (DeepSeek) which is provided with a structured prompt to assess the correctness of each verified triple. The LLM assesses each triple based on its accompanying supporting sentences and explanations, which offer contextual information to facilitate accurate evaluation. The LLM assigns a confidence score to each triple from a score range of 1–5, where 5 indicates strong confidence in the triple’s correctness and 1 denotes a high likelihood of error. This scoring system enables a more nuanced evaluation of triple correctness and facilitates differentiation between similar triples. Triples receiving confidence scores of 1 or 2 were excluded from the final SCS-KG to ensure high reliability. The defined score range reduces ambiguity in the LLM’s responses, encourages more definite assessments of the triples, and quantifies uncertainty regarding each triple. Each confidence score is supplemented with a rationale that explains the evaluation outcome, further enhancing the LLM’s reasoning ability and mitigating hallucination issues during the assessment process. The prompt for the entity and relation verification and the expected output format are illustrated in Table 2.

**Table 2:** Prompt for triple verification and output format.

Entity and Relation Verification Prompt	Expected Output Format
You are a professional knowledge graph builder. Based on the following information: **Supporting Sentences:** {supporting_sentences} **Explanation:** {explanation} Please determine whether the following triple is correct: **Head**:{head} **Relation**: {relation) **Tail**: {tail} Please rate the correctness of the triple, with the score ranging from 1 (least likely to be correct) to 5(most likely to be correct), and provide the reasons for the rating.	{ “**head**”: “南海文明(South China Sea Civilization)”, “**tail**”: “南海(South China Sea)”, “**relation**”: “存在 (exists)”, “**supporting sentence**”: “The South China Sea Civilization truly existed and still exists today; it has never been obliterated or illusory.”, “**explanation**”: “The text mentions that the South China Sea Civilization exists in the South China Sea region, indicating an existence relationship between the two.”, “**chunk ids**”: 2, “**result**”: “5”, “**reason**”: “The text states that the South China Sea Civilization truly existed and continues to exist, and that it is located in the South China Sea region. Based on the supporting sentences and explanations, it can be inferred that there is an ‘exists’ relationship between the two, hence the result is 5.” }

## 4. Experiments

### 4.1. Datasets and experiment setup

We compiled a corpus of raw textual data from 20 published Chinese books, 63 selected research articles sourced from the China National Knowledge Infrastructure (CNKI)-China’s largest comprehensive academic database-drawn from Peking University Core and Chinese Social Sciences Citation Index (CSSCI) journals, and official publications available from institutional websites (e.g., the Ministry of Foreign Affairs of the People’s Republic of China, the National Institute for South China Sea Studies, the Chinese Academy of Sciences, and the South China Sea Institute of Oceanology). All sources are publicly accessible through academic databases (e.g., CNKI), institutional websites, published books, or peer-reviewed journals, subject to standard access terms (e.g., institutional subscriptions for CNKI or library access for books). The collection and analysis methods complied fully with the terms and conditions of the respective sources, including CNKI’s usage policies for academic research purposes and the public-domain or fair-use provisions applicable to official government/institutional documents. No personal data or restricted content was collected or processed. The underlying raw texts include third-party copyrighted materials and content accessed under license (e.g., CNKI articles and published books) and therefore cannot be redistributed by the authors due to copyright and licensing restrictions. A complete list of sources is provided in the S1 Appendix to enable other researchers to independently obtain equivalent data. The corpus consists exclusively of Chinese-language materials, drawing primarily from official publications and academic sources affiliated with Chinese institutions. Accordingly, the resulting knowledge graph reflects the perspectives, terminology, and evidentiary emphases present within the selected corpus rather than a fully neutral representation of all international viewpoints concerning the South China Sea. After excluding irrelevant content such as references, appendices, copyright pages, and journal information, we constructed a corpus comprising 4,759,368 tokens. To handle this text, we use the RecursiveCharacterTextSplitter from Langchain configured with a chunk size of 500 tokens and 100-token overlap between chunks. These two values were chosen because preliminary experiments show that this combination preserves most sentence integrity while maximizing compatibility with the model’s context window length.

DeepSeek-V3 (https://platform.deepseek.com/) was chosen as the backbone model due to its outstanding proficiency in Chinese language processing and its effectiveness in knowledge-intensive tasks. As an open-source mixture-of-experts (MoE) model featuring 37 billion activated parameters, DeepSeek-V3 achieves superior performance on Chinese factual knowledge benchmarks, such as Chinese SimpleQA and C-Eval [36]. Owing to its robust reasoning capabilities and extensive training across diverse knowledge domains, DeepSeek-V3 is well-suited to effectively process and integrate the complex information necessary for knowledge graph construction. To ensure high accuracy and response consistency, the temperature of the LLM was set to 0.1.

For knowledge normalization, hierarchical clustering was performed using the average linkage method, with a similarity threshold of 0.85 applied for both cluster formation and subsequent merging of entities and relations. Entities and relations were grouped and consolidated if their cosine similarity with representative cluster element exceeded this threshold. The threshold of 0.85 was selected after inspecting clustering outcomes across a range of candidate values (0.80–0.95). Lower thresholds led to excessive merging of semantically distinct concepts (reduced cluster purity and increased false positives), while higher thresholds missed many legitimate synonym variants and variant expressions (lower recall and insufficient redundancy reduction). The value 0.85 achieved the optimal trade-off between precision and recall for both entities and relations.

To quantitatively assess extraction quality, we constructed a test set by randomly sampling 200 text segments from the corpus. Given the scale of manual annotation required for comprehensive entity and relation labeling, we employed a frontier LLM (GPT-5) as an independent annotator to produce initial gold-standard labels. GPT-5 is architecturally and operationally distinct from DeepSeek-V3 used in our extraction pipeline, thereby mitigating concerns of circular evaluation. To validate the reliability of this LLM-based annotation, we randomly selected 80 annotated samples for human review. The human review was conducted by the first author and a research assistant familiar with knowledge graph construction and South China Sea studies.

Both reviewers independently examined whether extracted entities, relations, and supporting evidence were consistent with the source text and semantically appropriate in context. Annotation quality was assessed based on the correctness of entity boundaries, entity types, relation labels, and factual consistency between triples and the corresponding source passages. Disagreements were subsequently resolved through discussion and consensus-based adjudication. The sampled human review yielded an entity-level accuracy of 89.7% and a triple-level accuracy of 85.4%. Discrepancies identified during human review were corrected to form the final gold standard containing 4811 entities and 4179 relation triples. We report Precision, Recall, and F1 at both the entity and triple levels, using relaxed matching (partial string overlap) to account for boundary ambiguity in Chinese text.

### 4.2. Main results

In this section, we present the results of entity and relation extraction, normalization of entities and relations, and triple verification.

#### 4.2.1. Results of entity and relation extraction.

**Fig 2:**
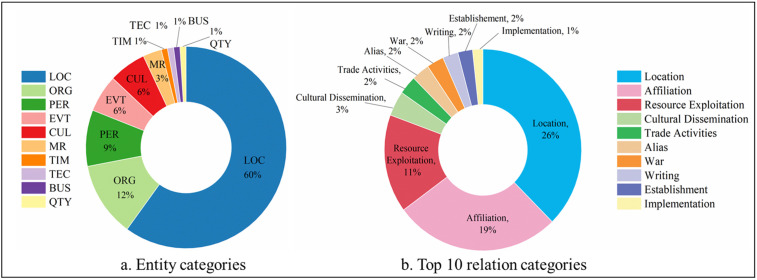
Entity and relation category distributions. (a) Entity type proportions showing the dominance of LOC entities (60%), followed by ORG (12%) and PER (9%); (b) Top relation types highlighting spatial (Geographical Location, 26%) and administrative (Affiliation, 19%) connections.

The entity and relation extraction process generated 78,542 nodes and 891,195 edges. As shown in Fig 2, location (LOC) entities dominate (60%), followed by organization (ORG) (12%) and person (PER) (9%). Other types such as quantity (QTY), business (BUS), technology (TEC), and time (TIM) each account for about 1%. A total of 894 distinct relation types were identified, reflecting the dataset’s diverse semantic structure. The most frequent relations include Geographical Location (26%) and Affiliation (19%), underscoring the centrality of spatial and administrative connections consistent with the predominance of LOC entities. Other major relations including Resource Exploitation (11%), Cultural Dissemination (3%), Trade Activities (2%), Alias (2%), War (2%), Writing (2%), Establishment (2%), and Implementation (1%) collectively indicate the multifaceted nature of the South China Sea domain, encompassing geopolitical, cultural, and economic dimensions.

Representative entities (Table 3) illustrate the breadth of extracted knowledge. LOC entities include Chinese provinces (e.g., *Hainan*, *Guangdong, Fujian*), neighboring states (*Vietnam*, *Philippines*, *Malaysia*, *Indonesia*), and other countries with strategic interests (*United States*, *Japan*, *United Kingdom*, *France*). Maritime features such as the *Nansha*, *Xisha*, *Zhongsha*, and *Dongsha Islands* serve as focal points of sovereignty disputes. The ORG category covers governmental bodies (e.g., *Qing government*), international and regional organizations (*UN*, *ASEAN*), military forces, and media institutions. EVENT entities span historical events (The Opium War, *1974 Xisha Sea Battle*), territorial disputes (*Huangyan Island dispute*), and international agreements (*Declaration on the Conduct of Parties in the South China Sea*). The PER category includes historical figures (*Zheng He*, *Emperor Wu of Han*), ethnic groups, and collectives (*Chinese fishermen*, *Huaqiao*). CULTURE entities encompass maritime relics, navigational texts, temples, religions, and art forms, while TIME, TECHNOLOGY, BUSINESS, and QUANTITY entities capture temporal markers, technological advancements, economic activities, and quantitative data relevant to the region.

**Table 3:** Entity categories and typical tokens.

Entity categories	Typical tokens
LOC	*South China Sea*, *China*, *USA*, *France*, *Japan*, *Xisha Islands*, *Vietnam*, *Nansha Islands*, *Philippines*, *Dongsha Islands*
ORG	*ASEAN*, *Qing government*, *Song government*, *United Nations*, *French government*
EVENT	*South China Sea arbitration case*, *Zheng He’s Navigation*, *Opium War*, *Sino- Vietnamese South China Sea conflict*, *Xisha naval battle*
PER	*Zheng He*, *Hainan fishermen*, *overseas Chinese*, *Chinese fishermen*
MR	*Oil*, *coral reefs*, *natural gas*, *spices*
CUL	*Genglubu*, *Buddhism*, *Chao Opera*, *Confucianism*, *Taoism*
TIM	*Song period*, *Song and Yuan periods*, *Sui and Tang*
TEC	*compass*, *lighthouse*, *watertight compartments*, *canoes*
BUS	*Overseas trade*, *tribute trade*, *Chinese merchant ships*, *trade circles*
QTY	*18 nautical miles*, *200 nautical miles*, *3.6 million square kilometers*

**Fig 3:**
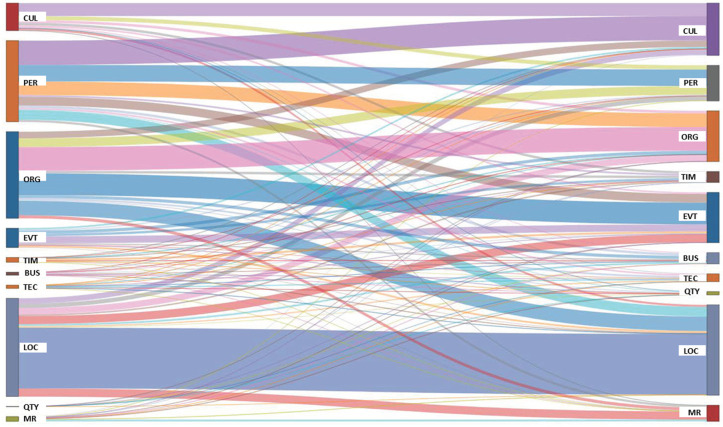
Pattern of co-occurring entity category pairs. LOC–LOC pairs are most common (strong spatial linkages); other frequent combinations (PER–CUL, ORG–ORG, ORG–EVT) highlight interpersonal, organizational, and cultural interactions.

Analysis of frequently co-occurring entity pairs (Fig 3) reveals that LOC-LOC pairs are the most common, reflecting strong spatial linkages. Other high-frequency combinations PER-CUL, ORG-ORG, ORG-EVT, and PER-PER highlight interactions among individuals, organizations, and cultural or event contexts. Examples include Fu *Hongguang*-*depicted*-*Map of Xisha and Nansha Islands* (PER-CUL) and *ASEAN 10 + 3*-*similar cooperation model*-*APEC* (ORG–ORG). Pairs such as ORG-LOC and PER-ORG reflect spatial or affiliative ties, while CUL-CUL and PER-LOC indicate cultural and locational associations. Overall, these patterns emphasize the prominence of geographical, organizational, interpersonal, and cultural relationships within the South China Sea knowledge structure.

#### 4.2.2. Results of entity and relation normalization.

**Fig 4:**
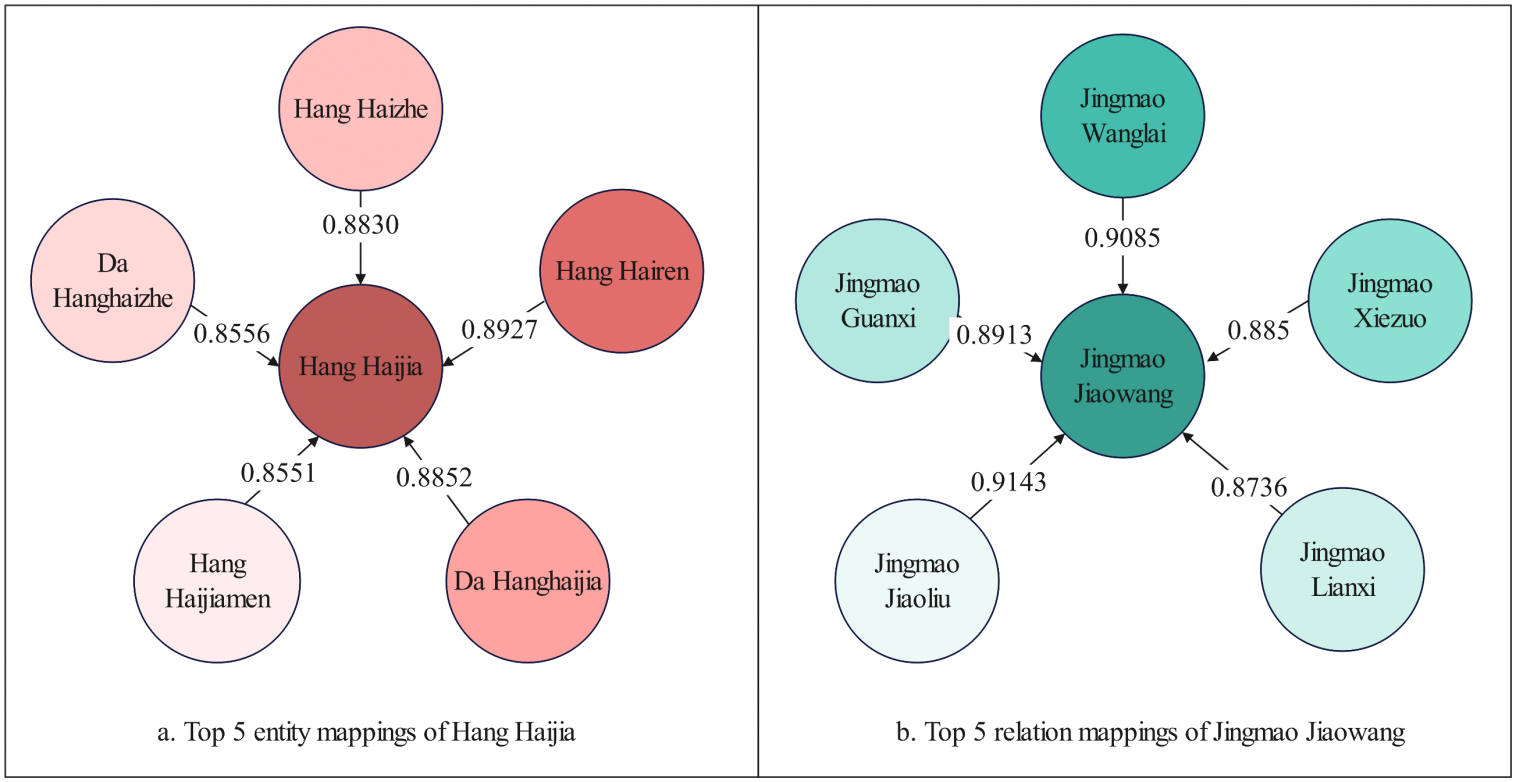
Examples of entity and relation mappings. (a) Entity cluster centred on *Hang Haijia* groups near-synonyms with high similarities (0.8551-0.8927); (b) Relation cluster centred on *Jingmao Wanglai* groups lexical variants with similarities 0.8736-0.9143.

After the normalization process, a total of 765,043 triples were generated. To ensure semantic consistency, both entities and relations underwent mapping based on similarity scores calculated by the LLM. The normalization stage effectively grouped linguistically or semantically related expressions, thereby reducing data redundancy while retaining meaningful distinctions among concepts (see Fig 4).

For entity mapping, the results indicate effective clustering of near-synonymous expressions (Fig 4a). For instance, the representative entity “南海周边国家” (countries surrounding the South China Sea) was semantically aligned with variants such as “南海周边有关国家” (relevant countries around the South China Sea, similarity 0.9338), “南海区域周边国家” (countries in the region surrounding the South China Sea, 0.8834), and “南海周边各国” (various countries around the South China Sea, 0.9332). Similarly, “南中国海问题” (the South China Sea issue) corresponded closely to “南中国海争端” (South China Sea dispute, 0.8676) and “中国南海问题” (China’s South China Sea issue, 0.8605), reflecting consistent clustering of conceptually similar entities. Another representative case is “200海里专属经济区” (200-nautical-mile exclusive economic zone), which mapped to several near-identical expressions, such as “200海里专属经济区域” (0.9816), “200海里经济专属区” (0.9256), and “200海里经济水域” (0.8805), illustrating the model’s precision in normalizing technical terms. Furthermore, named entities such as “乾隆皇帝” (Emperor Qianlong) were correctly linked to “乾隆帝” (0.9287) and “乾隆” (0.8937), indicating the model’s capacity to handle entity abbreviation and historical name variation.

In relation mapping, the normalization process also yielded coherent clusters of semantically similar expressions (Fig 4b). For example, the representative relation “被称为” (is called) was mapped to “被称作” (is called as, 0.9600), “被尊称为” (is honored as, 0.8512), and “被称之为” (is known as, 0.9596), reflecting high lexical and semantic overlap. Likewise, “宣称主权” (claim sovereignty) corresponded to “宣布主权” (declare sovereignty, 0.8985), “声称主权” (assert sovereignty, 0.8998), and “宣示主权” (proclaim sovereignty, 0.9236), accurately capturing lexical variations in expressing sovereignty claims. Other examples include “建立贸易关系” (establish trade relations), which aligned with “建立通商贸易关系” (0.9391) and “通商关系建立” (0.8625), as well as “和平解决纷争” (0.9498) and “和平解决争议” (0.9542) mapped to “和平解决争端” (peacefully resolve disputes).

Overall, the mapping results indicate that the normalization process effectively reduced lexical diversity without sacrificing semantic richness. High similarity scores (mostly above 0.85) suggest that the LLM successfully identified synonymous or near-synonymous expressions across both entities and relations. This normalization not only minimizes redundancy but also enhances the coherence and connectivity of the resulting knowledge graph, ensuring that conceptually equivalent terms and relationships are uniformly represented across the dataset.

#### 4.2.3. Results of triple verification.

**Fig 5:**
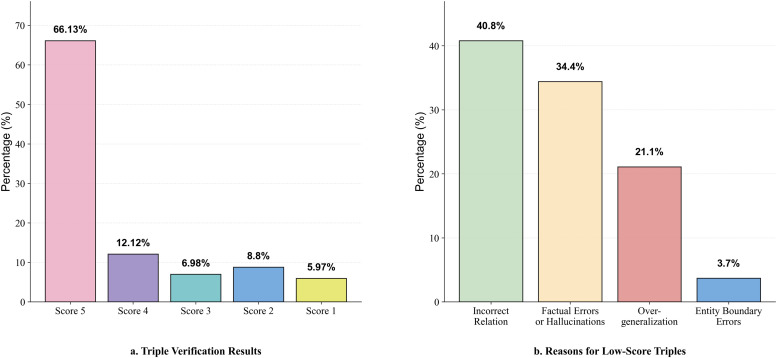
Triple verification results. (a) Confidence score distribution shows >78% of triples scored 4–5(high reliability); (b) Low-confidence triples (scores 1–2) primarily stem from incorrect relation classification (40.8%) and factual hallucinations (34.4%).

The verification procedure aimed to identify and reduce potential inaccuracies within the triples generated by the LLM. All triples from the normalization stage were re-evaluated for accuracy and consistency. Among the assessed triples, 505,896 (66.13%) received a confidence score of “5”, 92,760 (12.12%) scored “4”, 53,362 (6.98%) scored “3”, 67,341 (8.80%) scored “2”, and 45,684 (5.97%) scored “1”. Overall, more than 78% of the triples achieved high confidence levels (scores ≥ 4), indicating the robustness of the verification mechanism in enhancing data reliability (Fig 5a).

High-scoring triples were supported by explicit textual evidence, whereas medium- and low-scoring triples often reflected weaker contextual associations or implicit relationships. Further examination of low-confidence triples (scores 1–2) revealed four major error sources: **incorrect relation classification** (40.8%), **factual errors or hallucinations** (34.4%), **overgeneralization** (21.1%), and **entity boundary recognition errors** (3.7%) (Fig 5b).

Relationship misjudgment commonly occurred when the model incorrectly classified semantic links between entities, for example, interpreting “United Kingdom-Nansha Islands-geographical location” as a locational rather than exploratory relation. Factual errors often stemmed from fabricated or inconsistent details, such as the misrepresentation of “Eastern Xisha Islands” instead of “Xisha Islands”. Overgeneralization errors involved extrapolating conclusions beyond textual scope, as in extending the inapplicability of one legal framework to all similar systems. Finally, boundary errors arose when entity scopes were either too narrow or too broad, as seen in “South China Sea–geographical location–Subic of Philippines”, which improperly localized a regional reference.

Overall, the verification results suggest that the LLM-based validation framework effectively distinguishes reliable from erroneous triples, reduces semantic ambiguities, and enhances the overall accuracy and consistency of the knowledge graph.

#### 4.2.4. Quantitative evaluation.

To complement the structural and descriptive analyses presented above, we conducted a quantitative evaluation using the LLM(GPT-5)-annotated test set described in Section 4.1. Table 4 presents the extraction performance of our proposed method alongside two baseline approaches.

**Table 4:** Comparison of entity and relation extraction performance across different methods.

Method	Entity P	Entity R	Entity F1	Triple P	Triple R	Triple F1
SpaCy+OpenIE(B1)	0.6890	0.6188	0.6519	0.5608	0.5409	0.5507
GPT-4o(B2)	0.8923	0.8764	0.8843	0.8381	0.8254	0.8317
DeepSeek-V3(Ours)	0.8739	0.8934	0.8835	0.8599	0.8349	0.8472

As shown in Table 4, our DeepSeek-V3 based pipeline achieves competitive performance, with an entity-level F1 of 88.35% and a triple-level F1 of 84.72%. The traditional NLP pipeline (SpaCy+OpenIE) achieves noticeably lower scores, particularly on relation extraction (Triple F1 = 55.07%), reflecting the limitations of rule-based and statistical methods in handling domain-specific semantic complexity and long-range contextual dependencies in South China Sea texts. GPT-4o shows slightly higher entity-level precision but lower recall compared to DeepSeek-V3, suggesting a more conservative extraction strategy. In contrast, DeepSeek-V3 demonstrates stronger coverage in identifying heterogeneous entities and implicit semantic relations across historical, legal, and geopolitical materials, achieving the highest triple-level F1 (84.72%) among all evaluated methods.

### 4.3. Stage-wise evaluation

To further evaluate the contribution of each module, stage-wise experiments were performed on the entire framework. Table 5 presents core metrics used to assess the quality and structure of the knowledge graphs at three stages: Initial Extraction, Normalization, and Verification. These include: (1) **basic metrics** that quantify the graph’s scale and sparsity; (2) **connectivity metrics** that evaluate the extent of interconnection and component structure; and (3) **structural metrics** that analyze local and global topological properties. Basic metrics capture the graph’s size and connectivity density (see Table 5). The number of nodes and edges reflect the total counts of entities and relationships, respectively, while density characterizes sparsity according to the following formula:

**Table 5:** Knowledge graph quality metrics by stage.

Stage	Basic Metrics			Connectivity Metrics		Structural Metrics	
	Nodes	Edges	Density	Connected Components	LCC Ratio (%)^a^	Avg Clust Coeff^b^	Avg Shortest Path Len^c^
Initial Extraction	78,542	891,195	0.000 145	1523	76.32	0.135	4.28
Normalization	68,217	765,043	0.000 164	1214	82.54	0.168	3.75
Verification	59,836	652,018	0.000 183	982	87.46	0.192	3.32

^a^ LCC Ratio = Largest Connected Component Ratio.

^b^ Avg Clust Coeff = Average Clustering Coeﬀicient.

^c^ Avg Shortest Path Len = Average Shortest Path Length.


$$\begin{array}{c} \begin{array}{cc} & Density=\frac{\text{2}E}{N(N-\text{1})} \end{array} \end{array}$$ (2)

where *E* denotes the number of edges, and *N* denotes the number of nodes. Nodes and edges decreased from 78,542 and 891,195 (extraction stage) to 59,836 and 652,018 (after verification). At the same time, graph density rose from 0.000145 to 0.000183 (+26.2%). This reflects removal of redundant elements and greater compactness. Connectivity metrics evaluate how nodes form connected subgraphs. Key indicators are the number of connected components (disjoint subgraphs) and the Largest Connected Component (LCC) ratio (proportion of nodes in the largest subgraph). The LCC ratio is calculated as:


$$\begin{array}{c} \text{LCC }Ratio=\frac{\text{Nodes in LCC}}{N}\times \text{100}\% \end{array}$$ (3)

The initial extraction generated 1,523 components with a LCC ratio of 76.32%. Normalization merged small components, yielding 1,214 components and raising the LCC ratio to 82.54%. Verification reduced components to 982 and raised the LCC ratio to 87.46%, consolidating weak structures into the main component. Structural metrics assess both local clustering and global degree distribution within the graph. The average clustering coeﬀicient quantifies the extent to which a node’s neighbors are interconnected, and is defined for node *i* as $\frac{\text{2}E_{i}}{k_{i\;}\left(k_{i}-\text{1}\right)}$, where $E_{i}$ is the number of edges among neighbors and is the degree of node *i*. It increased from 0.135 (extraction) to 0.192 (verification), showing stronger local relationships. The average shortest path length reflects the eﬀiciency of connectivity among nodes. It decreased from 4.28 to 3.32, indicating better network eﬀiciency. This reduction may result from the elimination of redundant edges or from the normalization process, which prioritizes eﬀicient global connectivity over the formation of highly clustered local neighborhoods.

Degree centrality was also calculated to identify key entities across the three stages. Degree centrality quantifies the number of direct connections a node has, with higher values indicating greater centrality within the network. The degree centrality (C_D_) of node *i* in a graph with *n* nodes is given by:


$$\begin{array}{c} C_{D}(i)=\frac{k_{i}}{n-\text{1}} \end{array}$$ (4)

where $k_{i}$ represents the number of edges incident to node *i*. High degree centrality marks core concepts in the graph. Examination of the top 30 entities revealed that mapping successfully occurred during the normalization process. For example, entities such as “中国南海” (South China Sea), “南中国海” (South China Sea), and “中国南海海域” (South China Sea waters) were all mapped to “南海” (South China Sea), and “海南岛” (Hainan Island) was mapped to “海南” (Hainan). The node “南海” (South China Sea), which exhibited the highest degree centrality (normalized value = 1), was selected as the central node for constructing a local knowledge graph of the South China Sea corpus.

The subgraph was generated using the top 200 entities most strongly associated with “南海”. In the initial extraction stage, the subgraph exhibited a density of 0.156, indicating a moderate level of connectivity among these entities. An average clustering coeﬀicient of 0.56 indicated the presence of localized community structures, with nodes tending to form cohesive groups. The largest connected component ratio (1.0000) confirmed full connectivity within the subgraph, while an average shortest path length of 1.9015 reflected a “small-world” property, whereby most nodes were accessible within a few steps via “南海”(South China Sea) as the central hub.

Normalization improved density to 0.2031 (+30.2% from initial), raised clustering coefficient to 0.67, and slightly shortened average path length to 1.8970. Verification further adjusted density to 0.2001, increased clustering to 0.73, and reduced average path length to 1.61 (–15.3% from initial). This pronounced reduction reflects the retention of high-weight, semantically meaningful edges and the emergence of shortcut connections between distant communities, thereby facilitating more eﬀicient knowledge inference across domains.

To further quantify the individual contribution of each module, we conducted an ablation study by separately removing the normalization and verification stages while keeping all other settings unchanged. In addition to Entity F1 and Triple F1, we also introduce Cluster Purity as a semantic consistency metric. Cluster Purity measures the extent to which semantically related entities are grouped into coherent clusters after graph construction. Higher values indicate lower semantic redundancy and stronger conceptual consistency among connected entities.

Table 6 reports the results of the ablation study examining the contribution of each pipeline module. Removing the normalization module decreases Entity F1 from 88.35% to 84.10% and Triple F1 from 84.72% to 78.65%, while also lowering Cluster Purity from 0.92 to 0.79, Density from 0.000183 to 0.000145, and the LCC Ratio from 87.46% to 76.32%. These results indicate that normalization plays a critical role in merging synonymous entities, reducing semantic redundancy, and improving graph coherence and connectivity. Removing the verification module results in a smaller but still meaningful decline in Triple F1 from 84.72% to 81.25%, whereas Entity F1 remains relatively stable at 87.92%. This pattern is consistent with the verification module’s function as a relation-level quality filter that removes weakly supported or hallucinated triples. The decreases in Cluster Purity, Density, and LCC Ratio further suggest that verification improves both semantic consistency and structural reliability. Overall, the ablation results validate the effectiveness of the proposed three-stage pipeline, with normalization primarily enhancing semantic consistency and graph connectivity, and verification improving relational precision and factual reliability.

**Table 6:** Ablation study results showing the contribution of each pipeline module.

Configuration	Entity F1	Triple F1	Cluster Purity	Density	LCC Ratio
Full pipeline	88.35	84.72	0.92	0.000183	87.46
w/o Normalization	84.10	78.65	0.79	0.000145	76.32
w/o Verification	87.92	81.25	0.88	0.000164	82.54

### 4.4. Application scenarios

#### 4.4.1. Evidence-based query answering.

This scenario focuses on validating the effectiveness of the SCS-KG by addressing the complex, domain-specific query: “What historical evidence and claims concerning jurisdiction over the Nansha Islands and adjacent waters are represented in the SCS-KG corpus?” We compared responses generated under three conditions: DeepSeek alone, DeepSeek augmented with textual context, and DeepSeek integrated with the structured knowledge graph (DeepSeek+KG) to evaluate the KG’s ability to enhance response accuracy and contextual depth through structured domain knowledge. The following analysis is based on the selected source corpus represented in the SCS-KG and is intended to evaluate structured evidence retrieval rather than adjudicate competing sovereignty or jurisdictional claims.

**Fig 6:**
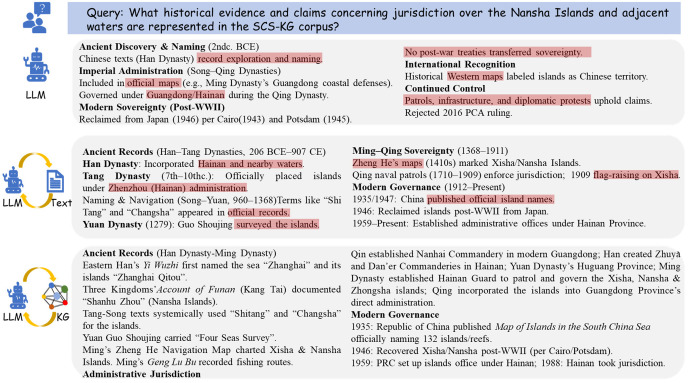
Answers generated under three scenarios. Red highlights indicate areas of vagueness, ambiguity, overgeneralization, or insufficient evidence grounding in the first two responses, in contrast to the more precise and source-linked output on the right.

The responses generated by the standalone DeepSeek model and the text-augmented DeepSeek model exhibited significant limitations (Fig 6). DeepSeek alone provided a broad chronological overview, often omitting specific dynastic details. The response was generic, relying on high-level references to official maps, and diminished in precision by vague or potentially confusing statements (as highlighted in red in Fig 6). For example, the response indicates that during the Qing period the islands were included in oﬀicial maps and administered under “Guangdong/Hainan”. Historical scholarship indicates that during the Qing Dynasty, the islands were consistently recorded in officially sanctioned imperial maps and administered under Guangdong Province within which Hainan was governed as the Qiongzhou Prefecture at that time [37]. Therefore, the reference to “Guangdong/Hainan” requires clarification, as Hainan was historically a prefecture under Guangdong until modern times, which could be confusing. Although the answer correctly notes that 1946 reclamation of territory from Japan based on the Cairo and Potsdam Declarations, the inclusion of the statement “No post-war treaties transferred sovereignty” lacks nuance and source attribution. According to historical and legal scholarship analyzing China’s position, both the Cairo Declaration and the Potsdam Proclamation are regarded by China as internationally significant instruments which explicitly stipulated the return of territories seized by Japan, forming a key post-war legal basis for China’s stated post-war legal position regarding the South China Sea islands [37,38].

Augmentation with general text offered some improvement in granularity but introduced new over-generalizations and potential inaccuracies (as highlighted in red). The DeepSeek+Textual Context response made unsubstantiated claims and overstated the evidentiary support for certain activities. Furthermore, ambiguous temporal references, such as the dual year reference for the publication of official island names, required clearer distinction. For instance, the assertion that the Tang Dynasty placed the islands under Zhenzhou (present-day Hainan) is not directly traceable to specific primary sources in the corpus. While some historical analyses and local records suggest this administrative allocation [39,40], contemporaneous primary Tang records concerning Hainan’s governance do not explicitly detail direct administrative control over the more distant South China Sea islands. Similarly, the statement that the Han Dynasty incorporated “Hainan and nearby waters” into its jurisdiction conflates distinct administrative scopes without source-level differentiation. Although the Han established commanderies like Zhuya and Dan’er on Hainan Island, extending this claim to constitute direct and sustained jurisdiction over specific South China Sea islands is an interpretation not fully substantiated by robust supporting evidence according to historical interpretations [37,41,42]. The claim that Guo Shoujing conducted specific astronomical surveys targeting the South China Sea islands is not substantiated by specific source references in the response. Although Yuan Dynasty historical records mention his “Sihai Ceyan” (Four Seas Survey) in the southern seas, direct and documented evidence pinpointing these activities to the Xisha or Nansha Islands remains limited and is partly based on later historical reconstructions [39]. Additionally, the reference to China publishing oﬀicial island names in “1935/1947” needs clarification. The Republic of China’s committee for the Review of Maps of Lands and Waters released a map designating island names in 1935 [40,43]. The reference to 1947 pertains to a post-World War II period when the Chinese Ministry of Internal Affairs officially renamed the islands and produced a location map of the South China Sea Islands [43,44]. A clearer distinction between these two separate official acts is necessary to avoid conflation.

**Fig 7:**
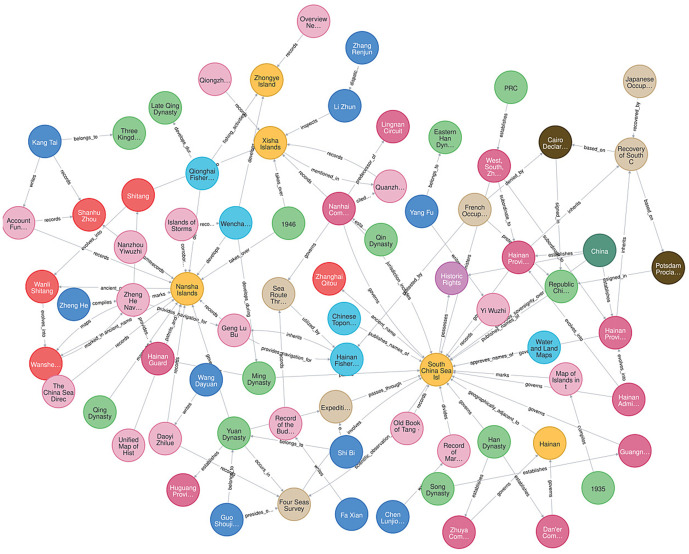
Subgraph for the query. The structured graph connects historical entities, events, primary sources, and multi-source evidence in a way that enables more source-grounded and contextually detailed reasoning and mitigates some of the generalization and precision issues seen in non-KG approaches.

By contrast, the DeepSeek+KG approach produced a more source-grounded and granular account. Leveraging the SCS-KG’s structured domain knowledge (Fig 7), this response systematically delineated historical records and jurisdiction-related claims represented in the corpus. It linked specific historical records and claims to explicit primary sources (e.g., Yi Wuzhi, Genglubu), concrete historical events, and clearly delineated administrative units. This structured approach produced responses that were more detailed and more consistently traceable to specific source texts contained within the corpus, helping reduce some of the oversimplification and generalization issues observed in the other two methods.

The case study indicates that the SCS-KG can integrate historical, geographical, and cultural data to provide more source-grounded and contextually detailed responses within the selected corpus context. The results suggest three key analytical strengths of the SCS-KG: Integrative Power (e.g., systematically linking fragmented data such as Yuan-era surveys to Ming-era navigation maps), Multi-Source Corroboration (e.g., linking Song-era administrative records with corresponding historical texts), and Nuanced Discovery (e.g., revealing implicit relationships between entities, such as the correlation between temple distributions and historical trade routes). By structuring domain knowledge into semantic triples and visual graphs, the SCS-KG supports a more systematic and nuanced investigation of complex interdisciplinary issues, demonstrating potential advantages over standalone language models in structured reasoning and evidence grounding, thereby supporting research use cases and potentially informing decision-support contexts.

#### 4.4.2. Implicit relationship discovery.

Historical narratives of the South China Sea rely on fragmented evidence, making the connections (i.e., latent, multi-hop relationships) between macro-level historical governance (e.g., imperial policies) and micro-level social practices (e.g., folk navigation, fishing) difficult to establish and visualize using conventional keyword-based document retrieval or linear archival research [27]. This analysis aims to address the following question: How can the SCS-KG be used to discover and visualize multi-hop evidence chains that connect macro-level historical governance with micro-level social practices?

To explore these latent linkages, we employed a constrained multi-hop path query approach [45], which identifies the shortest or all possible paths within a maximum hop length (κ) while filtering out semantically irrelevant connections. For this analysis, we set the maximum path length κ=5 to balance interpretive depth and network coherence.

**Fig 8:**
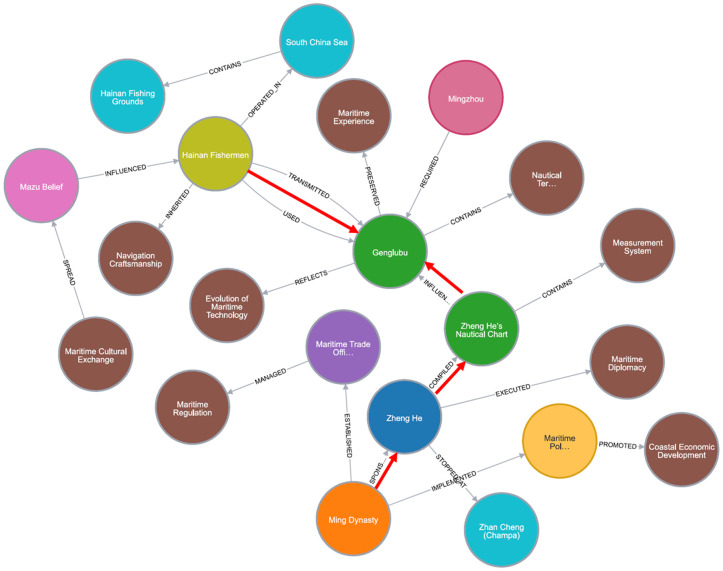
Relationship path between Ming Dynasty and Hainan Fisherman. The 5-hop chain links Ming state voyages to Hainan fishermen’s navigational practices, uncovering latent continuity in maritime knowledge that conventional methods struggle to reveal.

The query revealed several converging relational chains centered on Zheng He and the Genglubu through a coherent, 5-hop latent path, as illustrated in Fig 8. It connects state-level initiatives (the Ming Dynasty’s sponsorship of Zheng He’s voyages) with enduring grassroots practices (fishermen’s use of the Genglubu for navigation) through key intermediate Entities (Zheng He’s Nautical Chart and the Genglubu document). This structure illustrates how state-level initiatives and local navigational traditions are historically intertwined through the circulation and adaptation of maritime knowledge.

This result systematically integrates previously fragmented evidence across different levels of historical activity, constructing a novel, multi-level historical narrative demonstrating continuity in maritime knowledge and practice. The discovered path suggests the SCS-KG’s capacity to move beyond simple document retrieval and facilitate the reconstruction of complex, non-linear historical relationships.

#### 4.4.3. Entity-centric knowledge profiling.

Analyzing complex international relations and geopolitical processes requires the rapid, comprehensive profiling of key diplomatic instruments. This profiling depends on the structured aggregation of multi-dimensional information, including the identification of signatories (states/organizations), the underlying legal and institutional frameworks, core principles, spatial-temporal context, and subsequent developments or cooperative mechanisms. Traditional document-based approaches, however, remain linear, labor-intensive, and prone to information fragmentation and inconsistency across heterogeneous sources [46].

This scenario addresses the question: Can the SCS-KG serve as an analytical tool that enables “one-stop” aggregation and structured visualization of multi-dimensional information surrounding a major diplomatic agreement?

To address this, we adopted a 1-Hop Neighborhood Query approach, also known as an Ego-network Expansion. This graph-native method centers on a single entity (the ego node) and retrieves all one-hop neighbors (directly connected entities) and their semantic relationships (edges).The central entity (ego node) for this analysis is the *Declaration on the Conduct of Parties in the South China Sea* (*DOC*).

**Fig 9:**
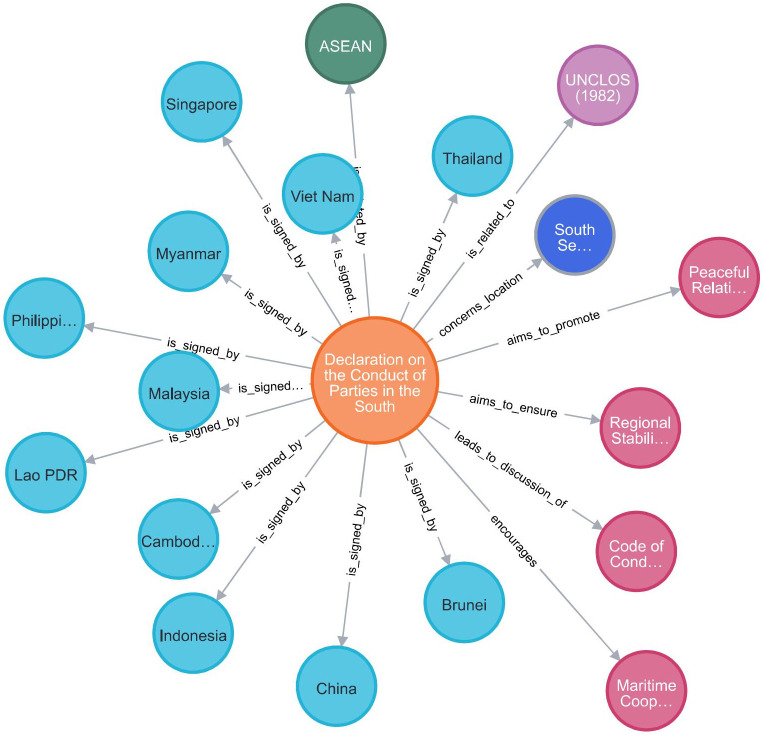
Structured Geopolitical Profiling of DOC. The visualization aggregates multidimensional elements (e.g., signatories, legal frameworks, objectives, and spatial context) into thematic clusters, illustrating SCS-KG’s efficiency for geopolitical analysis.

The query successfully generated a structured subgraph (Fig 9) that visually represents the core entity network of the DOC. Beyond showing connectivity, the visualization reveals distinct clusters that correspond to the agreement’s multidimensional nature. The first cluster (in light blue) encompasses the signatories, including China and the ASEAN member states, which collectively embody the multilateral character of the declaration. The second cluster (in purple) corresponds to the legal foundations, such as the 1982 United Nations Convention on the Law of the Sea (UNCLOS), which provides the normative and legal context for the DOC’s formulation. The third cluster (in pink) highlights the core objectives of the agreement, centering on notions of peaceful dispute resolution, mutual trust, maritime cooperation, and regional stability. The fourth cluster (in blue) shows that the South China Sea itself emerges as a distinct and central cluster, representing the spatial and geopolitical focus of the entire framework.

By categorizing these neighboring nodes, the SCS-KG not only aggregates information but also reveals the structural logic and thematic coherence of the DOC’s knowledge domain. The resulting entity-centric profile offers an integrated analytical perspective that connects actors, legal norms, policy goals, and institutional trajectories within a single visual frame. This suggests the SCS-KG’s potential as a rapid, structured, and comprehensive analytical instrument for geopolitical and policy research. By enabling instantaneous aggregation and contextualization of heterogeneous information sources, the graph significantly enhances analytical efficiency and provides a systematic foundation for the exploration of complex diplomatic processes.

## 5. Conclusion

This study developed and verified the SCS-KG through a three-stage LLM-assisted construction framework. The pipeline consisting of entity and relation extraction, entity and relation normalization, and triple verification facilitate the transformation of fragmented, multi-source information into a more structured and interpretable knowledge system. The initial extraction phase yielded 891,195 triples across ten entity categories, with a high concentration of spatial entities (LOC, 60%) and key relational pairs (LOC-LOC, PER-CUL, ORG-ORG) that reflect the region’s complex historical, cultural, and institutional fabric. Through knowledge normalization, 14.2% of triples were merged to standardize synonyms and variant expressions, and the triple verification process produced generally high reliability, with more than 78% of triples scoring 4 or 5 for accuracy.

The comprehensive evaluation of the constructed SCS-KG indicated its potential utility across three distinct application scenarios: evidence-based query answering, implicit relationship discovery and entity-centric knowledge profiling. Ablation and case studies indicated substantial improvements in graph density, connectivity, and real-world query answering performance. Specifically, the integration of DeepSeek and the SCS-KG consistently enhanced response accuracy and contextual depth, and achieved higher performance in the evaluated scenarios than standalone LLMs and LLMs utilizing only textual context. The integration of a generative model with a knowledge base can support more structured and source-grounded answers, offering explicit evidence chains while helping reduce factual hallucinations.

This work demonstrates the potential effectiveness of LLMs in supporting a modular KG construction process that integrates complex extraction, normalization, and verification. This framework contributes a potentially replicable workflow for other data-intensive and fragmented domains, such as environmental science, historical studies, and cultural heritage management. The constructed SCS-KG functions as a structured data resource for representing the South China Sea’s multifaceted aspects, contributing specifically to the development of domain-specific analytical resources by providing transparent evidence chains that enhance interpretability and reliability in future policy-relevant or historical research [2].

While the framework advances KG construction for the South China Sea, several limitations merit attention. The reliance on predominantly Chinese-language academic and official sources may introduce institutional and narrative biases, particularly in historical and legal interpretations of the South China Sea disputes. Consequently, the current SCS-KG should be interpreted as a corpus-grounded representation derived from the selected dataset rather than a politically neutral or exhaustive account of competing international perspectives. Additionally, while LLMs like DeepSeek-V3 enhance extraction efficiency, specialized sub-domains such as maritime law or archaeology may require further fine-tuning to accurately capture niche terminology. Because exhaustive manual validation of all triples is impractical at the current scale, the present study adopts sampled human review as a practical mitigation strategy rather than a full-scale expert validation procedure.

Future work will integrate multilingual corpora, including non-Chinese academic, legal, and archival sources, alongside broader expert-in-the-loop validation and cross-model verification for high-stakes triples, thereby improving cross-perspective balance, evaluation reliability, and factual precision within the SCS-KG. This will enable the KG to reflect ongoing regional developments more comprehensively and support balanced historical and policy analyses. By continuing to refine and expand this knowledge graph, we can facilitate more informed discussions on sovereignty, cultural heritage, and regional dynamics, supporting structured analysis and comparative examination of maritime governance narratives and international relations.

## Supporting information

S1 Appendix List of data sources. (DOCX)
