# Supplementary material for: Structured knowledge representation of the South China Sea: An LLM-based knowledge graph approach
Source: PLoS One. 2026 Jun 11;21(6):e0351132. doi: 10.1371/journal.pone.0351132 (PMC13257973; doi:10.1371/journal.pone.0351132)
Supplement: S1 Appendix — (DOCX) [file pone.0351132.s001.docx]

**S1 Appendix. List of data sources**

| Books | [1]安东尼•卡蒂.南海的历史与主权.新星出版社; 2023.  Antony Carty. The History and Sovereignty of the South China Sea Islands. New Star Press; 2023.  [2]戴胜德.中国南海海洋文化传.广东经济出版社; 2013.  Dai Shengde. Biography of China’s South China Sea Marine Culture. Guangdong Economy Press; 2013.  [3]寒冬.南海史话.广西师范大学出版社; 2011.  Han Dong. Acknowledge of South China Sea Series. Guangxi Normal University Press; 2011.  [4]韩振华.我国南海诸岛史料汇编.东方出版社; 1988.  Han Zhenhua. Compilation of Historical Materials on China’s South China Sea Islands. Oriental Publishing House; 1988.  [5]李德霞.南海领土争议中的媒体角色研究.厦门大学出版社; 2017.  Li Dexia. Research on the Role of Media in the South China Sea Territorial Dispute. Xiamen University Press; 2017.  [6]李国强.南中国海研究:历史与现状.黑龙江教育出版社; 2003.  Li Guoqiang. South China Sea Studies: History and Current Situation. Heilongjiang Education Press; 2003.  [7]李金明.中国南海疆域研究.黑龙江教育出版社; 2014.  Li Jinming. Researches on China’s South China Sea Coastal Areas and Territorial Seas. Heilongjiang Education Press; 2014.  [8]刘南威.中国南海诸岛史地研究论集.华南理工大学出版社; 2020.  Liu Nanwei. Collected Papers on the History and Geography of China’s South China Sea Islands. South China University of Technology Press; 2020.  [9]吕一燃.南海诸岛：地理、历史、主权.黑龙江教育出版社; 2012.  Lü Yiran. The South China Sea Islands: Geography, History, Sovereignty. Heilongjiang Education Press; 2012.  [10]浦野起央.南海诸岛国际纷争史[M].南京大学出版社; 2017.  Tatsuo Urano. South China Sea Territorial Dispute. Nanjing University Press; 2017.  [11]司徒尚纪，曹小曙，朱竑，许桂灵.环中国南海文化.商务印书馆; 2014  Situ Shangji, Cao Xiaoshu, Zhu Hong, Xu Guiling. Culture around the South China Sea. The Commercial Press; 2014.  [12]王崇敏.南海海洋文化研究.社会科学文献出版社; 2020.  Wang Chongmin. Research on South China Sea Marine Culture. Social Sciences Academic Press; 2020.  [13]吴士存.南海问题面面观（修订版）.时事出版社; 2016.  Wu Shicun. What One Needs to Know about the South China Sea. Current Affairs Press; 2016.  [14]吴士存.南海争端的由来与发展:南海纷争史国别研究.中华书局; 2022.  Wu Shicun. The Origin and Development of the Nansha Disputes: A Study on the History of South China Sea Issue from a Country-Specific Perspective. Zhonghua Book Company; 2022.  [15]新华社国际部.南海文明图谱:复原南海的历史基因.新华出版社; 2017.  International Department, Xinhua News Agency. From Junks to Galleons: Discovering the Civilization of the South China Sea. Xinhua Publishing House; 2017.  [16]杨翠柏.南沙群岛主权法理研究.商务印书馆; 2015.  Yang Cuibai. Legal Study on China’s Sovereignty over Nansha Islands. The Commercial Press; 2015.  [17]袁澍.中国与南海周边关系史.甘肃人民出版社; 2017.  Yuan Shu. History of Relations Between China and the Surrounding Areas of the South China Sea. Gansu People’s Publishing House; 2017.  [18]张海文.南海及南海诸岛.五洲传播出版社; 2014.  Zhang Haiwen. The South China Sea and its Islands. China Intercontinental Press; 2014.  [19]张一平、严春宝、林敏等.南海区域历史文化探微.暨南大学出版社; 2012.  Zhang Yiping, Yan Chunbao, Lin Min, et al. A Probe into the Regional History and Culture of the South China Sea. Jinan University Press; 2012.  [20][周伟民](https://book.douban.com/search/%E5%91%A8%E4%BC%9F%E6%B0%91)、[唐玲玲](https://book.douban.com/search/%E5%94%90%E7%8E%B2%E7%8E%B2).南海更路簿:中国人经略祖宗海的历史见证.海南出版社; 2016.  Zhou Weimin, Tang Lingling. The South China Sea Genglu Book: Historical Evidence of the Chinese Managing Their Ancestral Seas. Hainan Publishing House; 2016. |
| --- | --- |
| Journal articles | [1] 白天依.中国南海安全政策话语的演变与发展.当代外语研究, 2021, (02):53-63.  Bai, Tianyi. The evolution of security policy discourse on the South China Sea. *Contemporary Foreign Languages Studies*, 2021, (2): 53-63.  [2] 常娜.南海问题新特点与中国周边安全形势—2024年度“南海论坛”综述.亚太安全与海洋研究, 2025, (01):115-124+4. DOI:10.19780/j.cnki.ytaq.2025.1.7.  Chang, Na. New characteristics of the South China Sea Issue and neighborhood security of China—Overview of the 2024 South China Sea Forum. *Asia-Pacific Security and Maritime Affairs*, 2025, (1): 115-124+4. DOI:10.19780/j.cnki.ytaq.2025.1.7.  [3]陈进国.南海诸岛庙宇史迹及其变迁辨析.世界宗教文化,2015,(05):1-34+158.  Chen Jinguo. Analysis on the historical heritages and their changes of temples on Islands of the South China Sea. *World Religious Cultures,* 2015, (05): 1-34+158.  [4]陈俊霖,钱家英,王琦.关于南海诸岛研究的文献资料导引.图书馆学研究,2017,(22):97-101.DOI:10.15941/j.cnki.issn1001-0424.2017.22.015.  Chen, Junlin, Qian Jiaying, and Wang Qi. The guidance on literature of South China Sea Islands. *Research on Library Science*, 2017, (22): 97-101. DOI:10.15941/j.cnki.issn1001-0424.2017.22.015.  [5]戴正,郑先武.中国近年南海争端安全战略:“区别对待，双管齐下”.印度洋经济体研究,2019,(05):106-129+155-156. DOI:10.16717/j.cnki.53-1227/f.2019.05.007.  Dai, Zheng, and Zheng Xianwu. A preliminary discussion about China’s security strategy in the South China Sea Dispute in recent decade: “Treat Differently and Adopting Two-Pronged Strategy”. *Indian Ocean Economic and Political Review*, 2019, (5): 106-129+155-156. DOI:10.16717/j.cnki.53-1227/f.2019.05.007.  [6] 范子谦.历史主权与现实争端:改革开放以来大陆学界对南海主权的历史研究.中国海洋大学学报(社会科学版),2017,(03):13-22. DOI:10.16497/j.cnki.1672-335x.2017.03.003.  Fan, Ziqian. The history sovereignty and reality conflicts: A historical study on the sovereignty of the South China Sea since the Reform and Opening Up. *Journal of Ocean University of China (Social Sciences Edition)*, 2017, (3):13-22. DOI:10.16497/j.cnki.1672-335x.2017.03.003.  [7] 冯梁.南海战略形势：历史脉络与未来趋向.人民论坛·学术前沿,2021,(03):59-65. DOI:10.16619/j.cnki.rmltxsqy.2021.03.006.  Feng, Liang. Strategic situation in the South China Sea: Historical context and future trend. *Frontiers*, 2021, (3): 59-65.  DOI:10.16619/j.cnki.rmltxsqy.2021.03.006.  [8] 富宁宁,葛岳静,李彦征,等.南海周边国家对中国的对冲强度演变及对冲战略.地理学报,2024,79(08):2097-2114.  Fu, Ningning, Ge Yuejing, Li Yanzheng, et al. The evolution of hedging intensity and hedging strategies of countries surrounding the South China Sea towards China. *Acta Geographica Sinica*, 2024, 79 (8): 2097-2114.  [9] 郭荣星,杨书.南海划界争端：迈向新的地缘政治与经济均衡.经济地理,2019,39(10):22-28.DOI:10.15957/j.cnki.jjdl.2019.10.004.  Guo, Rongxing, and Yang Shu. The South China Sea Disputes: Toward a new geopolitical and economic equilibrium. *Economic Geography*, 2019, 39 (10): 22-28. DOI:10.15957/j.cnki.jjdl.2019.10.004.  [10] 郭声波.为什么说南海诸岛自古就是中国领土—10世纪以前中国对南海诸岛的发现、占有和管治.中国边疆史地研究,2023,33(01):142-154+216.  Guo, Shengbo. The reason that the South China Sea Islands have been Chinese territory since ancient times—China’s discovery, occupation and governance of the South China Sea Islands before the 10th century. *China’s Borderland History and Geography Studies*, 2023, 33 (1): 142-154+216.  [11] 侯丽维,张丽娜.全球海洋治理视阈下南海“蓝色伙伴关系”的构建.南洋问题研究,2019,(03):61-72.DOI:10.14073/j.cnki.nywtyj.2019.03.006.  Hou, Liwei, and Zhang Lina. The construction of “Blue Partnership” in the South China Sea from the perspective of global ocean governance. *Southeast Asian Affairs*, 2019, (3): 61-72. DOI:10.14073/j.cnki.nywtyj.2019.03.006.  [12] 胡波.当前南海局势的影响因素与走向分析.人民论坛·学术前沿,2021,(03):66-72.DOI:10.16619/j.cnki.rmltxsqy.2021.03.007.  Hu, Bo. Analysis of the influencing factors and trend of the current situation in the South China Sea. *Frontiers*, 2021, (3): 66-72.  DOI:10.16619/j.cnki.rmltxsqy.2021.03.007.  [13] 贾宇.南海航行自由:问题、规则与秩序.亚太安全与海洋研究,2019,(03):50-66+3.DOI:10.19780/j.cnki.2096-0484.2019.03.003.  Jia, Yu. A critical analysis on the FONOP in South China Sea. *Asia-Pacific Security and Maritime Affairs*, 2019, (3): 50-66+3. DOI:10.19780/j.cnki.2096-0484.2019.03.003.  [14] 李金明.中国是西沙、南沙群岛的最先发现者与开发者—评黎蜗藤《被扭曲的南海史:20世纪前的南中国海》.云南社会科学,2018,(04):1-8+186.  Li, Jinming. China is the 1st discoverer and developer of Xisha (Paracel) and Nansha (Spratly) Islands A Comment on Li Woteng’s Bei niu qu de Nan Hai shi: er shi shi ji qian de Nan Zhongguo Hai (*The Distorted History of SCS: SCS before the 20th Century*). *Social Sciences in Yunnan*, 2018, (4):1-8+186.  [15] 李锦辉.南海周边主要国家海底文化遗产保护政策分析及启示.太平洋学报,2011,19(06):72-84.DOI:10.14015/j.cnki.1004-8049.2011.06.002.  Li Jinhui. An analysis and revelation underwater culture heritage protection policy of the neighboring countries of the South China Sea. *Pacific Journal*, 2011, 19(06): 72-84. DOI:10.14015/j.cnki.1004-8049.2011.06.002.  [16] 李旷远, 阎根齐.历史时期南海诸岛命名考析.中州学刊, 2020, (10): 138-143.  Li, Kuangyuan, and Yan Genqi. On the naming of the South China Sea Islands in the historical period. *Academic Journal of Zhongzhou*, 2020, (10): 138-143.  [17] 李聆群.南海问题与大变局中的海上挑战.南京大学学报(哲学·人文科学·社会科学),2019,56(05):103-107.  Li, Lingqun. The South China Sea issue and maritime challenges in a time of great transformation. *Journal of Nanjing University (Philosophy, Humanities and Social Sciences)*, 2019, 56 (5):103-107.  [18] 李少丹.南海争端国际认知的塑造与再确认.南洋问题研究,2018,(04):17-28.DOI:10.14073/j.cnki.nywtyj.2018.04.002.  Li, Shaodan. International perception of South China Sea Dispute. *Southeast Asian Affairs*, 2018, (4): 17-28. DOI:10.14073/j.cnki.nywtyj.2018.04.002.  [19] 李永.论“南海更路簿”国际法上的证据资格——以苏德柳抄本“水路簿”等12种“南海更路簿”为样本.海南大学学报(人文社会科学版),2020,38(02):30-35.DOI:10.15886/j.cnki.hnus.2020.02.004.  Li, Yong. Assessing the competency of “Geng Lu Bu of the South China Sea”  in the evidence of the International Law. *Humanities and Social Sciences Journal of Hainan University*, 2020, 38(2): 30-35. DOI:10.15886/j.cnki.hnus.2020.02.004.  [20] 李忠林.中国对南海战略态势的塑造及启示.现代国际关系,2017,(02):23-30+66-67.  Li, Zhonglin. The strategic posture of China in the South China Sea and countermeasures. *Contemporary International Relations*, 2017, (2): 23-30, 66-67.  [21] 梁庭望.论中国人最早开发管理南海.中央民族大学学报(哲学社会科学版),2017,44(02):71-78.DOI:10.15970/j.cnki.1005-85  Liang, Tingwang. The Chinese People were the earliest inhabitants who developed and managed the South China Sea. *Journal of Minzu University of China (Philosophy and Social Sciences)*, 2017, 44 (2): 71-78. DOI:10.15970/j.cnki.1005-8575.2017.02.008.75.2017.02.008.  [22] 刘晨虹.中国南海历史性权利之“国际习惯法”说新解.太平洋学报,2019,27(09):1-12.DOI:10.14015/j.cnki.1004-8049.2019.09.001.  Liu, Chenhong. A new interpretation of the theory of “Customary International Law” on China’s historic rights in the South China Sea. *Pacific Journal*, 2019, 27(9): 1-12. DOI:10.14015/j.cnki.1004-8049.2019.09.001.  [23] 刘国良.论中国的南海海疆意识.海南大学学报(人文社会科学版),2019,37(06):23-29.DOI:10.15886/j.cnki.hnus.2019.06.004.  Liu, Guoliang. On China’s consciousness of the South China Sea maritime territory. *Journal of Hainan University (Social Sciences)*, 2019, 37 (6): 23-29. DOI:10.15886/j.cnki.hnus.2019.06.004.  [24] 刘琳.南海形势及发展趋势展望.亚太安全与海洋研究,2021,(02):72-90+3.DOI:10.19780/j.cnki.2096-0484.20210310.002.  Liu, Lin. The situation in the South China Sea and the prospect of its development. *Asia-Pacific Security and Maritime Affairs*, 2021, (2): 72-90+3. DOI:10.19780/j.cnki.2096-0484.20210310.002.  [25] 刘喆.南海问题的新形势与挑战—2020年度“南海论坛”综述.亚太安全与海洋研究,2021,(01):62-71+3.DOI:10.19780/j.cnki.2096-0484.20210108.001.  Liu, Zhe. The new situation and challenges of the South China Sea Issue: A summary of the 2020 ‘South China Sea Forum’. *Asia-Pacific Security and Maritime Affairs*, 2021, (1): 62-71+3. DOI:10.19780/j.cnki.2096-0484.20210108.001.  [26] 刘思瑞,王琦.南海争端语境下《更路簿》研究的新思考—以证据链为视角.太平洋学报,2023,31(02):95-106.DOI:10.14015/j.cnki.1004-8049.2023.02.008.  Liu, Sirui, and Wang Qi. New thoughts on the research of Genglubu in the context of the South China Sea Disputes—From the perspective of chain of evidence. *Pacific Journal*, 2023, 31 (2): 95-106. DOI:10.14015/j.cnki.1004-8049.2023.02.008.  [27] 刘天琦.全球海洋治理视域下的南海海洋治理.海南大学学报(人文社会科学版),2019,37(04):1-8.DOI:10.15886/j.cnki.hnus.2019.04.001.  Liu, Tianqi. Ocean governance in the South China Sea from the perspective  of global ocean governance. *Humanities and Social Sciences Journal of Hainan University*, 2019, 37 (4): 1-8. DOI:10.15886/j.cnki.hnus.2019.04.001.  [28] 刘文波.南海地缘政治格局与海上丝绸之路建设的地缘战略选择.理论与现代化,2016,(03):35-39.  Liu, Wenbo. The geo-political options of South China Sea geo-political pattern and 21st century Maritime Silk Road. *Theory and Modernization*, 2016, (3): 35-39.  [29] 刘云刚,刘玄宇,张争胜.渔民视角下中国南海的领域构建.地理科学,2020,40(07):1062-1071.DOI:10.13249/j.cnki.sgs.2020.07.003.  Liu, Yungang, Liu Xuanyu, and Zhang Zhengsheng. The territorial structure in  South China Sea from fishermen’s perspective. *Scientia Geographica Sinica*, 2020, 40 (7): 1062-1071.  DOI:10.13249/j.cnki.sgs.2020.07.003.  [30] 楼春豪.地区海洋秩序视角下的南海问题.太平洋学报,2017,25(11):58-68.DOI:10.14015/j.cnki.1004-8049.2017.11.006.  Lou, Chunhao. South China Sea Issue in the perspective of regional maritime order. *Pacific Journal*, 2017, 25 (11):58-68. DOI:10.14015/j.cnki.1004-8049.2017.11.006.  [31] 罗亮,吴士存.中国构建南海国际话语权：实践探索与未来进路.东北亚论坛,2024,33(01):50-63+127.DOI:10.13654/j.cnki.naf.2024.01.004.  Luo, Liang, and Wu Shicun. China constructs international discourse power on the South China Sea: Practical exploration and future path. *Northeast Asia Forum*, 2024, 33 (1): 50-63, 127. DOI:10.13654/j.cnki.naf.2024.01.004.  [32] 栗广.新中国成立以来中国南海维权方式的演进.党史研究与教学,2020,(03):34-44.  Li, Guang. Evolution of the way of safeguarding rights in the South China Sea since the founding of new China. *CPC History Research and Teaching*, 2020, (3): 34-44.  [33] 庞卫东.南海争端:阶段、特点及成因.史学月刊,2019,(04):133-136.  Pang, Weidong. The South China Sea Dispute: Stages, characteristics, and causes. *Journal of Historical Science*, 2019, (4): 133-136.  [34] 祁怀高.近年来中国在南海的存在格局、面临挑战及因应之策.国际论坛,2018,20(01):8-13+79.DOI:10.13549/j.cnki.cn11-3959/d.2018.01.002.  Qi, Huaigao. China’s presence and challenges in the South China Sea in recent years China’s SCS policies in the future. *International Forum*, 2018, 20 (1): 8-13+79. DOI:10.13549/j.cnki.cn11-3959/d.2018.01.002.  [35] 宋培军.海洋法公约“历史性”话语体系及其南海历史权利认知意义.思想战线,2020,46(01):126-139.  Song, Peijun. “Historicity” discourse in the United Nations’ Convention on the Law of the Sea and its import to the South China Sea. *Thinking*, 2020, 46 (1): 126-139.  [36] 孙冬虎.南海诸岛外来地名的命名背景及其历史影响.地理研究,2000,(02):217-224.  Sun, Donghu. The background and influence of the exotic toponyms in the South China Sea Islands. *Geographical Research*, 2000, (2): 217-224.  [37] 涂少彬.全球治理视阈下南海安全合作机制的建构.法商研究,2019,36(06):163-176.DOI:10.16390/j.cnki.issn1672-0393.2019.06.014.  Tu, Shaobin. Construction of a security cooperation mechanism in the South China Sea from the perspective of global governance. *Studies in Law and Business*, 2019, 36 (6): 163-176. DOI:10.16390/j.cnki.issn1672-0393.2019.06.014.  [38] 王崇敏, 阎根齐.南海海洋历史与文化研究的新成果.中国海洋大学学报(社会科学版),2016,(02):81-83. DOI:10.16497/j.cnki.1672-335x.2016.02.012.  Wang, Chongmin, and Yan Genqi. The new research achievements of the history and culture of the South China Sea. *Journal of Ocean University of China (Social Sciences Edition)*, 2016, (2): 81-83. DOI:10.16497/j.cnki.1672-335x.2016.02.012.  [39] 王崇敏,阎根齐,刘亮.《更路簿》发现和研究40年.中国史研究动态,2018,(06):38-47.  Wang, Chongmin, Yan Genqi, and Liu Liang. Discovery and study on Manual of Sea Routes (Geng Lu Bu) during the past 40 years. *Trends of Recent Researches on the History of China*, 2018, (6): 38-47.  [40] 王灵桂.南海问题中十个不容改变的基本事实.太平洋学报,2016,24(07):47-50+63.  Wang, Linggui. Ten facts that can not be changed in the South China Sea Issue. *Pacific Journal*, 2016, 24 (7): 47-50+63.  [41] 王巧荣.20世纪80年代中国的南海政策.当代中国史研究,2016,23(02):25-34+124-125.  Wang, Qiaorong. Chinese policy of the South China Sea in the 1980s. *Contemporary China History Studies*, 2016, 23 (2): 25-34+124-125.  [42] 王晓鹏,郑海麟.中国对南海诸岛主权的关键性历史依据与战略选择.太平洋学报,2016,24(07):57-60+63.  Wang, Xiaopeng, and Zheng Hailin. Critical historical basis and strategic choice of China on the South China Sea Islands sovereignty. *Pacific Journal*, 2016, 24 (7): 57-60+63.  [43] 吴建树.论中国在南海政策的转变.亚太安全与海洋研究,2019,(02):98-111+4.DOI:10.19780/j.cnki.2096-0484.2019.02.007.  Wu, Jianshu. On China’s transition on the South China Sea policy. *Asia-Pacific Security and Maritime Affairs*, 2019, (2): 8-111+4. DOI:10.19780/j.cnki.2096-0484.2019.02.007.  [44] 吴士存.南海缘何再度成为大国角逐的舞台.人民论坛·学术前沿,2021,(03):16-27.DOI:10.16619/j.cnki.rmltxsqy.2021.03.003.  Wu, Shicun. Why has the South China Sea become a competing arena for the great powers? *Frontiers*, 2021, (3): 16-27.DOI:10.16619/j.cnki.rmltxsqy.2021.03.003.  [45] 夏代云.论中国海南渔民对南海岛礁及海域的历史性所有权.海南大学学报(人文社会科学版),2016,34(04):11-17.DOI:10.15886/j.cnki.hnus.2016.04.002.  Xia, Daiyun. On the historical ownership of the fishers in Hainan for the South China Sea Islands and reefs and its waters. *Journal of Hainan University (Social Sciences)*, 2016, 34 (4): 11-17.  DOI:10.15886/j.cnki.hnus.2016.04.002.  [46] 徐晓东.南海问题所涉历史依据英文研究述评.太平洋学报,2020,28(10):70-79.DOI:10.14015/j.cnki.1004-8049.2020.10.006.  Xu, Xiaodong. A review of English literature on historical evidence of South China Sea Issue. *Pacific Journal*, 2020, 28 (10): 70-79. DOI:10.14015/j.cnki.1004-8049.2020.10.006.  [47] 徐晓天.南海形势稳中有忧.现代国际关系,2017,(12):23-24.  Xu, Xiaotian. The South China Sea situation: Stable but with concerns. *Contemporary International Relations*, 2017, (12): 23-24.  [48] 阎根齐.论海南渔民的航海技术与中国对南海的历史性权利.吉林大学社会科学学报,2019,59(02):195-204+224.DOI:10.15939/j.jujsse.2019.02.ls3.  Yan, Genqi. On the maritime technology of Hainan fishermen and China’s historic rights over the South China Sea. *Jilin University Journal of Humanities and Social Sciences*, 2019, 59 (2): 195-204, 224. DOI:10.15939/j.jujsse.2019.02.ls3.  [49] 阎根齐.论南海海上丝绸之路的形成时间.学术探索,2017,(03):133-137.  Yan, Genqi. On the formation time of the Maritime Silk Route on Nanhai. *Academic Exploration*, 2017, (3): 133-137.  [50] 叶淑兰.南海地缘战略逻辑演进与南海命运共同体建构.世界地理研究,2022,31(04):687-699.  Ye, Shulan. Evolution of geostrategic logic of the South China Sea and construction of a community of shared future in the South China Sea. *World Regional Studies*, 2022, 31(4): 687-699.  [51] 尹继武.中国南海安全战略思维:内涵、演变与建构.国际安全研究,2017,35(04):33-61+155.DOI:10.14093/j.cnki.cn10-1132/d.2017.04.003.  Yin, Jiwu. China’s strategic thinking toward South China Sea: Concept, evolution and construction. *Journal of International Security Studies*, 2017, 35 (4): 33-61+155. DOI:10.14093/j.cnki.cn10-1132/d.2017.04.003.  [52] 于文杰,朱静怡,徐桑奕.民国时期地图绘制及南海诸岛指称的历史演变.学术研究,2016,(02):119-125.  Yu Wenjie, Zhu Jingyi, Xu Sangyi. The historical evolution of map drawing and the designation of the South China Sea Islands during the Republic of China period. *Academic Research*, 2016, (02): 119-125.  [53] 张静焕,李永平.历史性权利视角下南海问题的法理分析.海南大学学报(人文社会科学版),2017,35(02):23-27.  DOI:10.15886/j.cnki.hnus.2017.02.003.  Zhang, Jinghuan, and Li Yongping. A legal analysis of the South China Sea Issues from the perspective of historic rights. *Journal of Hainan University (Social Sciences)*, 2017, 35 (2): 23-27. DOI:10.15886/j.cnki.hnus.2017.02.003.  [54] 张乃根.中国对南海诸岛屿领土主权的一般国际法依据.甘肃社会科学,2017,(06):122-128.DOI:10.15891/j.cnki.cn62-1093/c.2017.06.020.  Zhang, Naigen. The General International Law basis for China’s territorial sovereignty over the South China Sea Islands. *Gansu Social Sciences*, 2017, (6): 122-128. DOI:10.15891/j.cnki.cn62-1093/c.2017.06.020.  [55] 张朔人,张若城.南海维权的民间证据——《更路簿》内涵与面世抄本研究.云南师范大学学报(哲学社会科学版),2018,50(04):26-35.  Zhang, Shuoren, and Zhang Ruocheng. Historical evidence for our safeguarding the rights of the South China Sea: A study of the implications of the book Genglubu and its different handwritten versions. *Journal of Yunnan Normal University (Humanities and Social Sciences Edition)*, 2018, 50 (4): 26-35.  [56] 张志洲.南海问题上的话语博弈与中国国际话语权.探索与争鸣,2020,(07):126-134+160.  Zhang, Zhizhou. Discourse game and China’s international discourse power on the South China Sea Issue. *Exploration and Free Views*, 2020, (7): 126-134+ 160.  [57] 张玉强.“21世纪海上丝绸之路”倡议下中国南海话语权的提升.和平与发展,2019,(06):70-86+133-134.  Zhang, Yuqiang. Enhancing China’s discourse power over the South China Sea under the “21^st^ Century Maritime Silk Road” Initiative. *Peace and Development*, 2019, (6): 70-86+133-134.  [58] 赵昌平,郑米雪,范厚明.“南海问题”响应的复杂网络演化进程分析.中国软科学,2016,(08):1-16.  Zhao, Changping, Zheng Mixue, and Fan Houming. The analysis of complex network evolution process about “South China Sea Issue” response. *China Soft Science*, 2016, (8): 1-16.  [59] 郑漫,程志敏.“南海文明”刍议—以历史地理学和地缘政治学的视角.海南大学学报(人文社会科学版),2022,40(06):62-69.DOI:10.15886/j.cnki.hnus.202109.0820.  Zheng, Man, and Cheng Zhimin. Preliminary remarks on the South China Sea civilization from the perspective of historical geography and geopolitics. *Journal of Hainan University (Social Sciences)*, 2022, 40 (6): 62-69. DOI:10.15886/j.cnki.hnus.202109.0820.  [60] 朱锋.大变局下的南海局势：新问题与新特点.人民论坛·学术前沿,2021,(03):45-58. DOI:10.16619/j.cnki.rmltxsqy.2021.03.005.  Zhu, Feng. The situation in the South China Sea amid the great changes: New problems and new features. *Frontiers*, 2021, (3): 45-58. DOI:10.16619/j.cnki.rmltxsqy.2021.03.005.  [61] 朱锋.南海局势的新动向、新态势、新挑战.亚太安全与海洋研究,2025,(03):1-20+133. DOI:10.19780/j.cnki.ytaq.2025.3.1.  Zhu, Feng. New dynamics, new trend and new challenges of the South China Sea situation. *Asia-Pacific Security and Maritime Affairs*, 2025, (3): 1-20+133. DOI:10.19780/j.cnki.ytaq.2025.3.1.  [62] 邹克渊,刘昕畅.南海仲裁案与中国在南海的历史性权利.东南亚研究,2017,(04):92-113+155-156.DOI:10.19561/j.cnki.sas.2017.04.092.  Zou, Keyuan, and Liu Xinchang. South China Sea Arbitration Case and China’s entitled historic rights in the South China Sea. *Southeast Asian Studies*, 2017, (4): 92-113+155-156. DOI:10.19561/j.cnki.sas.2017.04.092.  [63] 左希迎.南海秩序的新常态及其未来走向.现代国际关系,2017,(06):33-40+63-64.  Zuo, Xiying. The new order in South China Sea and its trends. *Contemporary International Relations*, 2017, (6): 33-40+63-64. |
| Websites | <https://scsio.cas.cn/xisha/zyts/rwls/202503/t20250324_7563588.html>  <https://igsnrr.cas.cn/news/cmsm/202011/t20201102_5730265.html>  <https://www.nanhai.org.cn/index.php/Index/Research/review_c/id/157.html>  <https://www.mfa.gov.cn/web/zyxw/201607/t20160713_338590.shtml>  <https://www.mfa.gov.cn/web/zyxw/201607/t20160712_338577.shtml>  <https://www.mfa.gov.cn/web/ziliao_674904/zcwj_674915/200011/t20001122_7949769.shtml>  <https://scsio.cas.cn/xisha/zyts/zrdl/202503/t20250324_7563593.html>  <https://scsio.cas.cn/xisha/zyts/202503/t20250317_7558785.html>  <https://scsio.cas.cn/xisha/zyts/zrdl/202503/t20250324_7563591.html>  <https://scsio.cas.cn/xisha/zyts/202503/t20250314_7553902.html>  <https://scsio.cas.cn/xisha/zyts/rwls/202503/t20250324_7563587.html> |
